# Development of Holistic Face Processing From Childhood and Adolescence to Young Adulthood in Chinese Individuals

**DOI:** 10.3389/fpsyg.2020.00667

**Published:** 2020-04-09

**Authors:** Yini Sun, Qinglan Li, Xiaohua Cao

**Affiliations:** Department of Psychology, Zhejiang Normal University, Jinhua, China

**Keywords:** face, composite effect, development, holistic processing, cognitive

## Abstract

Previous studies have indicated that holistic face processing is important for the development of face perception. The purpose of this study was to verify the development trajectory of holistic processing, from older childhood to young adulthood, using the complete composite paradigm. Participants from three different age groups (children, adolescents, young adults) were recruited for this study. The results showed that all groups demonstrated the composite effect with similar magnitude. Furthermore, face processing performance improved with age. These results, together with previous results, imply it is a race-general phenomenon that holistic face processing is similar among older children, adolescents, and young adults.

## Introduction

Over the past few decades, several lines of behavioral and neural studies have led to the consensus that human face perception is a type of visual expertise (Diamond and Carey, 1986; Harel, 2016) that features holistic processing (Gauthier et al., 2003; Richler and Gauthier, 2014), i.e., the combination of various facial features into a gestalt (Rossion, 2013). More generally, holistic processing is a perceptual strategy for piecing together fragmented information; it is highly automated due to extensive exposure of the member of a particular objects category (Wong and Gauthier, 2010; Richler et al., 2012). The holistic processing strategy used in face perception has been most frequently explored through the composite face paradigm (Young et al., 1987; Hole, 1994; Farah et al., 1998). In this paradigm, the top and bottom halves of different faces are combined to a new “composite” face. Participants are asked to attend to the top (or bottom) half of a composite face while ignoring the bottom (top) half. Performance is better on congruent than incongruent trials in the aligned condition, indicating that participants have difficulty ignoring the unattended part; the congruency effect is larger in the aligned than misaligned condition; this has been termed the face composite effect (Young et al., 1987; Hole, 1994; Richler et al., 2008; Wong et al., 2011).

Developmental studies on the face composite effect have shown that young children have an adult-like ability for holistic face processing (Carey and Diamond, 1994; Durand et al., 2007; Mondloch et al., 2007; Meinhardt-Injac et al., 2017; Petrakova et al., 2018; Ventura et al., 2018). For example, Meinhardt-Injac et al. (2017) investigated holistic face processing beginning from childhood to older adulthood and showed that children have an adult-like holistic face processing ability by the age of 10. Using emotional faces, Durand et al. (2007) also found that, by the age of 11, children exhibit a composite face effect that is similar to that of adults. In an earlier study, Carey and Diamond (1994) presented composite faces from familiar and unfamiliar faces to children and required them to name the top halves of the composite faces. A face composite effect was observed in 10-year old children.

Apart from such face composite studies, previous research has also revealed that the overall face processing ability appears to gradually improve during childhood and reaches an adult-like level by late adolescence (see McKone et al., 2012). For instance, Lawrence et al. (2008) examined 500 young people aged 6–16 years and found a linear relationship between age and face processing performance. Germine et al. (2011) traced the ability to recognize new faces from pre-adolescence through middle age and found that face-learning ability continues to improve until just after the age of 30.

To summarize, previous studies have shown that holistic processing of faces reaches an adult-like level by the age of 11; however, the other aspects of face processing performance continue to develop until adulthood. On a surface level, it appears that holistic processing does not contribute to the development of face processing abilities from older childhood to adulthood. Several researchers have suggested that the development of face processing abilities from older childhood to adulthood is the result of general improvement of various cognitive abilities (e.g., attention), rather than the mastering of holistic processing strategy (Crookes and Mckone, 2009; McKone et al., 2012). If this is indeed true, it is reasonable to infer that the ability of holistic processing remains stable from older childhood to adulthood. Unfortunately, there is little evidence that supports this theory. To our best knowledge, only one recent study has investigated this issue. In this study, Petrakova et al. (2018) recruited participants from ages 6 to 21 and assessed their performance using a complete composite face paradigm. The results showed that the composite face effect was not influenced by age; in other words, holistic processing reached an adult-like level in young children. Importantly, the ability of holistic face processing did not change much from older childhood to adulthood.

The participants in the study of Petrakova et al. (2018) were all Caucasians. Holistic processing of faces is also present in Asian adults (Hayward et al., 2008; Li et al., 2017, 2019; Wang et al., 2019) and importantly, the level of holistic face processing appears higher in Asians than in Caucasians (Chua et al., 2005; Lewis et al., 2008; Miyamoto et al., 2011). For example, Miyamoto et al. (2011) recruited adult Japanese and American participants to study configural processing of faces and found that Japanese adults performed better. Previous studies have also shown that the ability to process unfamiliar faces is higher in East Asian than in Western adults (Michel et al., 2006a; Mondloch et al., 2010; Crookes et al., 2013). Using different face tasks (e.g., the inversion task in Rhodes et al., 1989; the composite face task in Michel et al., 2006b; and the whole-part task in Tanaka et al., 2004), the evidence also consistently showed that for unfamiliar faces (i.e., other-race faces), the ability of holistic face processing (i.e., the composite effect) of Asian participants was better than that of Caucasian participants. Overall, the level of holistic face processing is higher in adult East Asians than in adult Caucasians. As noted, in Caucasians, the holistic face processing level stabilized from childhood, since the age of 11 years, to adulthood. Unfortunately, the developmental trajectory of holistic face processing in East Asians of the same age range is unclear. With the evidence noted above, it is reasonable to infer that there are two possibilities regarding the development trajectories of holistic face processing from the age of 11 years until adulthood in East Asians. The first possibility is that by age 11, East Asian children may have holistic face processing levels similar to those of Caucasians of the same age; thus, East Asians continue to develop this ability, which eventually leads to a higher level of holistic processing into adulthood. The second possibility is that, by age 11, even younger East Asian children may have higher levels of holistic face processing than Caucasians of the same age, and may have reached an East Asian adult-like level, after which it remains relatively stable until adulthood. Namely, the holistic face processing level from age 11 to adulthood in East Asians may stabilize. The present study thus aimed to verify the two possibilities by examining holistic face processing in East Asians from late childhood (10–12 years old) to young adulthood (20–24 years old).

In the present study, a complete composite face paradigm similar to that of Petrakova et al. (2018) was used to assess holistic face processing in Chinese children, adolescents, and adults. To our best knowledge, two versions of the composite face task with different indexes of holistic face processing were used—the partial and the complete versions. The partial version indicated that the attended parts of the two faces are either same or different, whereas the unattended halves are always different. Holistic face processing was defined as the alignment effect (i.e., the difference between aligned and misaligned conditions). In the complete version, both the attended and unattended halves were either the same or different, which yielded the “congruency” condition between the critical and irrelevant halves. Several studies demonstrated that there are differences between the two versions of the composite face paradigm (Richler et al., 2012; Rossion, 2013; Richler and Gauthier, 2014). First, the two versions are based on different meanings of holistic face processing; the partial design was mapped onto global face templates, but the complete design was termed as inflexible attentional weightings (Richler et al., 2012). Second, the indexes computed with the two versions yielded qualitatively different results with similar designs (De Heering et al., 2007; Ventura et al., 2018). For example, De Heering et al. (2007) used the partial design and found that holistic face processing emerged by the early age of four, whereas Ventura et al. (2018) employed the complete version and showed that children reached an adult-like level of holistic face processing at the age of six, which indicates the difference between the two measurements. Third, the measurement of partial design is susceptible to strategy and response bias (Richler and Gauthier, 2014). Because of these reasons and with a paradigm similar to that of Petrakova et al. (2018), our study applied the complete composite face paradigm.

To summarize, Petrakova et al. (2018) confirmed that the holistic face processing ability of Western individuals remains similar from older childhood to adulthood. Moreover, the evidence indicates that holistic face processing abilities differed in Western and Eastern participants. This raises the issue of whether the holistic face processing ability from older childhood to adulthood in Eastern participants has a similar trajectory to the previous development studies of Western participants; in other words, is the developmental trajectory race-specific or race-general? Hence, the present study recruited Chinese people from three different age groups (children aged 10–12 years, adolescents aged 16–18 years, and young adults aged 20–24 years) and used the complete composite paradigm to address the issue.

## Materials and Methods

### Participants

We used G power to estimate the sample size, with 95% power and η_*p*_^2^ ranging from 0.26 to 0.38. We needed a sample size of 12–25 participants in each group [the power and η_*p*_^2^ was derived from a meta-analysis study (Richler and Gauthier, 2014)]. Thus, 75 participants were recruited, comprising three age groups: 25 children (aged 10–12 years, Mean = 10.3 ± 0.5, 14 females), 25 adolescents (aged 16–18 years, Mean = 16.9 ± 0.6, 18 females) from local schools, and 25 young adults from Zhejiang Normal University (aged 19–24 years, Mean = 20.2 ± 1.1, 14 females; see Supplementary Material). All participants were native Chinese speakers, and reported being right-handed. They had normal or corrected-to-normal vision, and reported no psychiatric or neurological disorders. The participants were paid for their time, and it was ensured that none of them were familiar with the experimental design. Written informed consent was obtained from all participants (or their parents) before the experiment began. The research protocols reported in the present study were approved by the ethical committee of Zhejiang Normal University.

### Materials

**FIGURE 1:**
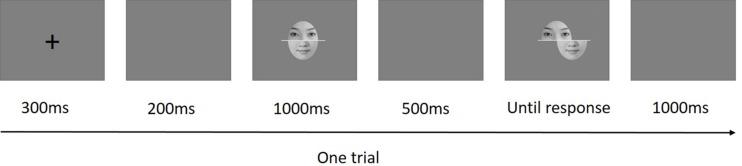
Example of a misaligned trial used in the present experiment. Observers were instructed to ignore the bottom half of the study face, and indicate whether the top half of the test face was the same as that of the study face. The faces were always studied in the aligned format, and test faces were either presented in the aligned format or misaligned format.

Twenty gray-scale pictures of Chinese faces (10 females) were selected from a set of faces used in a previous research conducted in our laboratory (Cao et al., 2015). All faces used in the present study had neutral facial expressions; their external features (such as hair, glasses, and ears) were removed and replaced by the same oval contour using Adobe Photoshop CS5 (San Jose, CA, United States). Thereafter, to create composite faces, a three-pixel-thick horizontal white line was positioned in the middle of the nose to divide the original face into two halves. Thereafter, the top half (i.e., forehead) was combined with the bottom half (i.e., mouth) of another same sex face, thereby creating a new composite face (Young et al., 1987). All composite faces were 300 × 280 pixels, and subtended 4.5° × 4° from a viewing distance of 60 cm. Each composite face had two versions: aligned, in which the top and bottom halves were aligned to form a standard face template; and misaligned, in which the bottom halves were shifted to the right by 60 pixels of face width. The top half was always presented in the center of the screen, regardless of whether the composite face was aligned or misaligned (see Figure 1).

### Procedure

The participants were required to sit in a dimly lit room at a distance of 60 cm from 17-inch cathode ray tube (CRT) monitors (60 Hz, 1024 × 768 pixel resolution). All stimuli were presented against a dark gray background (R: 128, G: 128, B: 128) using E-Prime 2.0 software (Psychology Software Tools, Pittsburgh, PA, United States).

Each trial began with a fixation cross presented at the center of the screen for 300 ms, followed by a dark gray blank screen for 200 ms. Next, the study face was presented for 1000 ms, followed by a dark gray blank for 500 ms. Finally, the test face was presented on the screen until the participants responded. The inter-trial interval was 1000 ms. This procedure is shown in Figure 1. Participants were instructed to judge whether the top halves of the composites were the same or different while ignoring the bottom halves of the composite face. Participants were asked to respond as accurately and quickly as possible by pressing the corresponding keys. When the top halves were identical, half of the participants were asked to press “A” with their left hands, and press “L” with their right hands when the top halves were different; for the other half, the key pressing requirement was reversed.

The experiment contained 160 trials that were divided randomly into four blocks. Each block consisted of 10 trials for each of the four conditions (Alignment × Congruency). The four cells of the composite paradigm were congruent-aligned, congruent-misaligned, incongruent-aligned, and incongruent-misaligned. In the misaligned trials, the first composite face was aligned, whereas the second was misaligned. In the aligned trials, both were aligned composite faces^1^. In the congruent condition, the top and bottom halves of the test faces were either the same as that of the study face, or both halves were different from that of the study face; however, in the incongruent condition, the corresponding top halves of the study and test faces were the same and the corresponding bottom halves were different (or vice versa). In the congruent-aligned condition, the two halves of the study and test faces were aligned. In addition, the attended and unattended halves for the study and test faces were either the same or different. In the congruent-misaligned condition, the study and test face halves (i.e., attended and unattended halves) were either the same or different when the test faces were misaligned. In the incongruent-aligned condition, the corresponding top halves of the study and test faces were the same and the corresponding bottom halves were different (or vice versa) when the test faces were aligned. In the incongruent-misaligned condition, the corresponding top halves of the study and test faces were the same and the corresponding bottom halves were different (or vice versa) when the test faces were misaligned.

A 3 (Group: children, adolescents, young adults) × 2 (Alignment: aligned, misaligned) × 2 (Congruency: congruent, incongruent) mixed design analysis of variance (ANOVA) model was used, with a between-factor of group and repeated-measures factors of congruency and alignment. The four cells of the composite paradigm are congruent-aligned, congruent-misaligned, incongruent-aligned, and incongruent-misaligned. The participants were made to practice 16 trials to understand the procedure before engaging in the formal experiment. The stimuli used in the practice stage were not used in the formal experiment. Participants took a short break after finishing each block. The total duration of the experiment was approximately 10 min.

### Data Analysis

Three children and five adolescents were excluded from further analysis because of not following the instructions (two adolescents) or chance performance (three children and three adolescents). Therefore, the final sample sizes of each group were 22 children, 20 adolescents, and 25 undergraduates. For the trials, we excluded participants whose correct reaction times exceeded 5000 ms and were lower than 200 ms. Additionally, the trials with more than Mean ± 3 SD for correct reaction time were also excluded (Zhao et al., 2013). The trials excluded were no more than 3% of the total.

The analysis measures were mean sensitivity (A’) and response time (RT). A’ represents response sensitivity for each condition according to the signal detection theory. Sensitivity is widely used and relatively unaffected by response bias when the assumptions of normality and equal variances are violated (Stanislaw and Todorov, 1999; Wong et al., 2012; Tso et al., 2014; Chung et al., 2018). Therefore, it is appropriate for evaluating the pure composite face effect. A’ was computed using the following formula:

$$A\prime =0.5+sign\left(H-F\right)\left[\frac{{\left(H-F\right)}^{2}+\left|H-F\right|}{4\max\left(H,F\right)-4HF}\right]$$

In this formula, *H* represents the hit rate (the proportion of same responses to same trials), and *F* refers to the false alarm rate (the proportion of same responses to different trials). The response time was calculated as the correct reaction time between the onset of the test stimuli and the participant’s response. In the present study, we expected there to be interactions between the congruency and alignment in the three groups, and there is a three-way interaction among the group, congruency, and alignment that implies that holistic face processing develops from childhood to adulthood.

## Results

### Sensitivity (A’)

**FIGURE 2:**
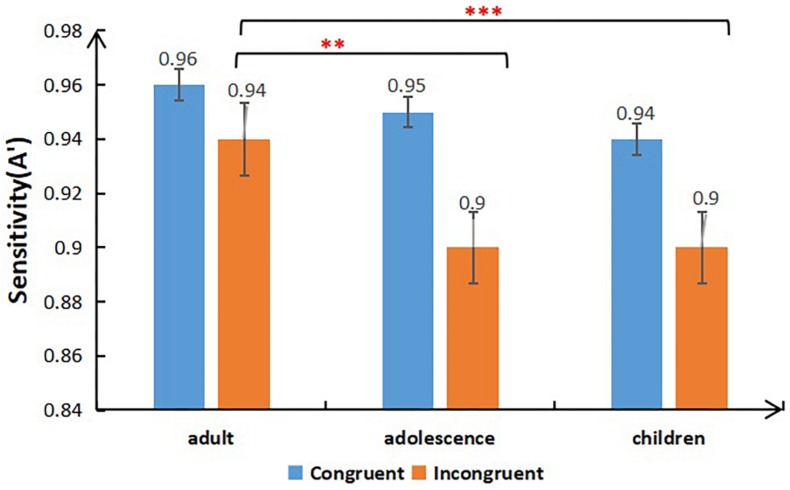
Mean sensitivity (A’) for congruent and incongruent trials as a function of Group (adults/adolescents/children) (**p < 0.01; ***p < 0.001).

**FIGURE 3:**
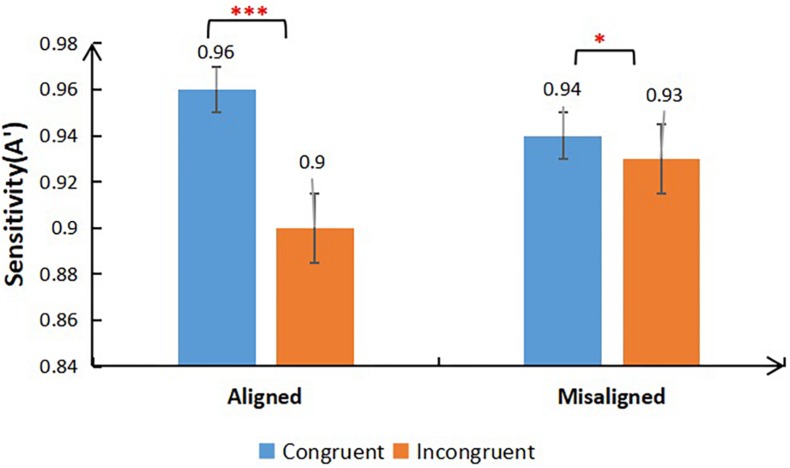
Mean sensitivity (A’) for congruent and incongruent trials as a function of Alignment (aligned/misaligned) (*p < 0.05; ***p < 0.001).

The results of sensitivities (A’) are presented in Table 1 and Figures 2, 3. A 3 × 2 × 2 mixed ANOVA was conducted on sensitivity (A’), with Group (children, adolescents, young adults) as the independent-groups variable, and Congruency (congruent, incongruent) and Alignment (aligned, misaligned) as the repeated-measures variables. The analysis revealed a significant main effect of Congruency [*F*_(__1,64__)_ = 59.685, *p* < 0.001, η_*p*_^2^ = 0.483], whereby sensitivity in the congruent condition was significantly higher than in the incongruent condition. There was a significant main effect of Group [*F*_(__2,64__)_ = 5.673, *p* = 0.005, η_*p*_^2^ = 0.151]. Moreover, a significant interaction was found between Congruency and Group [*F*_(__2,55__)_ = 4.982, *p* = 0.010, η_*p*_^2^ = 0.153]. The *post hoc* independent-samples *t-*tests revealed that there were no significant differences among the groups in the congruent condition. Critically, in the incongruent condition, the face sensitivities of adults were significantly greater than those of adolescents [*t*_(__43__)_ = 3.275, *p* = 0.002, *Cohen’s d* = 0.84] and children [*t*_(__45__)_ = 4.138, *p* < 0.001, *Cohen’s d* = 1.33]. There was no difference between the face sensitivities of adolescents and children [*t*_(__40__)_ = 0.544, *p* = 0.589, *Cohen’s d* = 0.2], which indicates the presence of continuous development of face processing ability from adolescence to young adulthood.

**TABLE 1:** Mean sensitivity (A’) for holistic face processing.

	Aligned		Misaligned	
	Congruent	Incongruent	Congruent	Incongruent
Adults (*n* = 25)	0.97 ± 0.02	0.93 ± 0.05	0.95 ± 0.05	0.95 ± 0.05
Adolescents (*n* = 20)	0.96 ± 0.05	0.89 ± 0.08	0.94 ± 0.06	0.91 ± 0.06
Children (*n* = 22)	0.95 ± 0.03	0.89 ± 0.04	0.94 ± 0.04	0.92 ± 0.04

Importantly, the results revealed a significant interaction between Congruency and Alignment [*F*_(__1,64__)_ = 26.096, *p* < 0.001, η_*p*_^2^ = 0.290]. Further analysis using paired-samples *t*-tests revealed that in the aligned condition, sensitivity was significantly greater in the congruent trials than in incongruent trials [*t*_(__66__)_ = 8.000, *p* < 0.001, *Cohen’s d* = 1.26]. In the misaligned condition, sensitivity was significantly greater in the congruent trials than in the incongruent trials [*t*_(__66__)_ = 2.020, *p* = 0.047, *Cohen’s d* = 0.2]. This result indicated that the face composite effect was observed in each group (see Supplementary Material). Importantly, there were no three-way interactions, which implies that the magnitude of holistic face processing among the three groups was similar. Due to the present study being based on the absence of a three-way interaction between Group, Congruency, and Alignment, we turned to Bayesian analyses to provide an index of the strength of evidence for the absence of differences in configural processing of faces in the three groups. The Bayesian analyses of the three-way interaction between Group, Congruency, and Alignment demonstrated very weak evidence for a significant interaction (H1), BF10 = 0.122, but strong evidence for the null hypothesis (H0), BF01 = 7.946 (see Morey et al., 2016). These results, along with those from conventional ANOVA, establish that there are no differences in the configural processing of faces in the three groups. The analysis was performed using JASP^2^.

### Response Time

A 3 × 2 × 2 mixed ANOVA was performed on the correct response times (Table 2). The results revealed a significant main effect of Congruency [*F*_(__1,64__)_ = 17.628, *p* < 0.001, η_*p*_^2^ = 0.216], whereby the RTs of congruent trials were faster than those of incongruent trials. The main effect of Alignment was also significant [*F*_(__1,64__)_ = 34.394, *p* < 0.001, η_*p*_^2^ = 0.350]; the RTs for aligned faces were faster than those of misaligned faces. In addition, a significant main effect of Group was found [*F*_(__2,64__)_ = 23.090, *p* < 0.001, η_*p*_^2^ = 0.419].

**TABLE 2:** Mean response times (ms) for holistic face processing.

	Aligned		Misaligned	
	Congruent	Incongruent	Congruent	Incongruent
Adults	778 ± 154	843 ± 180	841 ± 163	820 ± 151
Adolescents	620 ± 99	653 ± 104	675 ± 114	664 ± 100
Children	925 ± 187	970 ± 206	1016 ± 214	1000 ± 210

The interaction between Alignment and Group was significant [*F*_(__2,64__)_ = 6.182, *p* = 0.004, η_*p*_^2^ = 0.162]. *Post hoc t*-tests revealed that in the aligned condition, the RTs of adolescents were significantly faster than those of adults [*t*_(__43__)_ = 3,945, *p* < 0.001, *Cohen’s d* = 1.21] and children [*t*_(__40__)_ = 6.424, *p* < 0.001, *Cohen’s d* = 2.02], whereas the RTs of children were slower than those of adults [*t*_(__45__)_ = 2.974, *p* = 0.005, *Cohen’s d* = 0.86]. Furthermore, in the misaligned condition, the RTs of adolescents were also significantly faster than those of adults [*t*_(__43__)_ = 3.491, *p* = 0.001, *Cohen’s d* = 1.07] and children [*t*_(__40__)_ = 6.711, *p* < 0.001, *Cohen’s d* = 2.11], whereas the RTs of children were slower than those of adults [*t*_(__45__)_ = 3.931, *p* < 0.001, *Cohen’s d* = 1.14].

Importantly, similar to the result of sensitivity (A’), Congruency × Alignment was also significant [*F*_(__1,64__)_ = 29.864, *p* < 0.001, η_*p*_^2^ = 0.318]. *Post hoc t*-tests revealed that in the aligned condition, the RTs in the congruent trials were faster than those in the incongruent trials [*t*_(__66__)_ = 6.588, *p* < 0.001, *Cohen’s d* = 0.26]; however, in the misaligned condition, there was no significant difference between the congruent and incongruent conditions [*t*_(__66__)_ = 1.717, *p* = 0.091, *Cohen’s d* = 0.06]. There were no three-way interactions, which implies that the magnitude of holistic face processing among the three groups was similar. As the present study was based on the absence of a three-way interaction between Group, Congruency, and Alignment, we used Bayesian analyses to provide an index of the strength of evidence for the absence of differences in configural processing of faces in the three groups. The Bayesian analyses demonstrated very weak evidence for a significant interaction (H1), BF10 = 0.34, but moderate evidence for the null hypothesis (H0), BF01 = 2.997 (see Morey et al., 2016). These results, along with those from conventional ANOVA, establish that there are no differences in the configural processing of faces in the three groups.

## Discussion

The present study used a complete composite face paradigm to examine the developmental trajectory of holistic face processing from older childhood to young adulthood. The results revealed a similar composite face effect (holistic processing) among the three groups, which indicates that the holistic face processing ability had already reached adult-like levels by the age of 11. The results directly verify that the holistic face processing ability from older childhood to adulthood is similar in Eastern individuals.

The finding is in accordance with those of previous studies, such as Durand et al. (2007), who indicated that a similar composite face effect was observed in both 11-year-old children and young adults. Furthermore, Xiao et al. (2017) investigated the effect of facial movements on holistic face processing in Chinese children, adolescents, and adults using a composite face task. They indicated that in the static face condition, 12-year-old children exhibited a composite effect similar to that of adults. To our best knowledge, only one recent study used a complete composite paradigm to investigate the development of holistic face processing. In that study, the authors also demonstrated that the holistic face effect remained relatively constant from middle childhood to young adulthood in German individuals (Petrakova et al., 2018). Using the complete composite paradigm, our study extended the finding that the holistic face effect is relatively constant from childhood to young adulthood in Chinese people. This indicates that there is cross-cultural consistency in the development of the composite face effect from middle childhood to young adulthood.

Interestingly, in the present study, face processing performance improved with age, particularly in the incongruent condition. Prior studies have indicated that the face processing performance of adults is considerably better than that of children (De Heering et al., 2007; Mondloch et al., 2007), and that of older adults (Meinhardt-Injac et al., 2014), especially in the incongruent condition. This is also consistent with prior indications that face processing performance develops with age (see Germine et al., 2011 for a review; McKone et al., 2012; Megreya and Bindemann, 2015) and with the general cognitive development theory of Crookes and Mckone (2009). The theory emphasizes that face perception fully matures early in life and that improvement in face processing tasks after early age is entirely due to the development of general cognitive factors (Crookes and Mckone, 2009; McKone et al., 2012), such as memory ability and speed of neural processing. Previous studies have also reported that these general cognitive abilities continue to develop into early adulthood (Plude et al., 1994; Adleman et al., 2002; Schroeter et al., 2004). Importantly, our results extend the general cognitive development theory derived from the Western people to the Chinese people, indicating that the theory indicates consistency across the races.

Interestingly, our results showed that the response times of both adolescents and adults were faster than those of children, whereas adolescents’ response times were faster than those of adults. Although this outcome was unexpected, it is supported by previous studies, such as that of Lin et al. (2016), which demonstrated that the response times and accuracies of teenagers (13–19 years) were better than those of young adults (20–24 years). This phenomenon can probably be explained by the development of the prefrontal cortex (PFC). Previous studies have demonstrated that the density of spines on the pyramidal cells in the PFC decreases between adolescence and adulthood (Mrzljak et al., 1990). Furthermore, other studies have demonstrated that the degree of PFC development can influence an individual’s executive functioning (Burgess et al., 2000). In the present study, the adolescents’ response times were faster than those of adults, which is inconsistent with the results of Petrakova et al. (2018). In the research of Petrakova et al. (2018), the adolescents’ response times were not different from those of adults, and there were cues on the test face in their study, but not in our study. Without cues, the participants may be more focused on the attended halves. The adolescents may have a reaction advantage over the adults in the complete composite face task without changing attention in each trial. Therefore, future studies should investigate this issue.

There are some limitations of the present study that must be addressed in future research. First, the present study used Chinese adult faces as stimuli, which have been used to elicit the other-age effect reported in previous studies (Susilo et al., 2009; Hills and Lewis, 2018). Future studies using age-related Chinese faces may help us to further explore the development of face processing in Chinese participants. Second, we found an adult-like holistic face processing ability among children aged 10–12 years. However, some studies have found a similar ability among those aged 4–6 years (De Heering et al., 2007; Mondloch et al., 2007; Ventura et al., 2018). For example, Ventura et al. (2018) demonstrated that the holistic face processing emerges in children by the age of 6 years in Caucasians. The present study just indicated that the Chinese children aged 11 years have the similar face holistic processing ability as do adults. The developmental trajectory of holistic face processing before the age of 11 years is unknown in Chinese children. Further studies should assess young Chinese children to directly verify the age of emergence of holistic processing and the earliest age of reaching the adult-like level, as did in Ventura et al. (2018). Finally, the face materials used in the present study were presented repeatedly, which can affect the holistic face measurement. For example, Richler et al. (2015) correlated the Cambridge Face Memory Test with the composite face task. They indicated that the correlation was mediated by the face repetition. That is, the correlation was significant only when the face parts were repeated, but not when the face parts did not repeat. Since the face repetition may affect the measurement of holistic face processing, further studies should adopt faces that are presented only once to investigate forward.

## Conclusion

The present study reported a similar composite effect from older childhood to adulthood in Chinese persons, which indicates that there is no cultural variation between Eastern and Western individuals in this respect.

## Data Availability Statement

The datasets generated for this study are available on request to the corresponding author.

## Ethics Statement

The studies involving human participants were reviewed and approved by the ethical committee of Zhejiang Normal University. Written informed consent to participate in this study was provided by the participants’ legal guardian/next of kin. Written informed consent was obtained from the individual(s), and minor(s)’ legal guardian/next of kin, for the publication of any potentially identifiable images or data included in this article.

## Author Contributions

QL and XC designed the experiments. YS, QL, and XC performed the data analysis and wrote the manuscript.

## Conflict of Interest

The authors declare that the research was conducted in the absence of any commercial or financial relationships that could be construed as a potential conflict of interest.

## Footnotes

1 The standard manipulation of misaligned trials in previous studies (e.g., Rossion, 2013) was that both the first and second faces were misaligned. In the present study, the manipulation of misaligned trials with sight different from the standard manipulation of misalignment.

2 https://jasp-stats.org
