# Supplementary material for: Development of Holistic Face Processing From Childhood and Adolescence to Young Adulthood in Chinese Individuals
Source: Front Psychol. 2020 Apr 9;11:667. doi: 10.3389/fpsyg.2020.00667 (PMC7161039; doi:10.3389/fpsyg.2020.00667)
Supplement: Supplementary file 1 [file Data_Sheet_1.PDF]

Supplementary Materials

1 The Results of each group

1.1 Sensitivity (A') in each group

A 2 (Congruency: congruent, incongruent) × 2 (Alignment: aligned, misaligned) repeated-measures ANOVA was conducted on each group separately (see Figure 4). For adults, there was a significant main effect of congruency ($F_{(1, 24)} = 7.879$, $p = .01$, $\eta_p^2 = .247$), with the sensitivity in the congruent condition higher than that in the incongruent condition. The interaction between Congruency and Alignment was significant ($F_{(1,24)} = 10.141$, $p = .004$, $\eta_p^2 = .297$). The post-hoc paired-samples *t*-tests revealed that in the aligned condition, sensitivity in the congruent trials was higher than that in the incongruent trials ($t_{(24)} = 4.037$, $p < .001$, *Cohen's d* = 1.16), whereas in the misaligned condition, the difference between the congruent and incongruent trials was not significant ($t_{(24)} = 0.622$, $p = .54$, *Cohen's d* = 0.28).

For adolescents, the main effect of congruency was significant ($F_{(1, 19)} = 19.625$, $p < .001$, $\eta_p^2 = .508$). The sensitivity in the congruent condition was higher than that in the incongruent condition. There existed Congruency × Alignment interaction ($F_{(1,19)} = 7.530$, $p = .013$, $\eta_p^2 = .284$). The post-hoc paired-samples *t*-tests showed that in the aligned condition, the sensitivity in the congruent trials was higher than in the incongruent trials ($t_{(19)} = 4.731$, $p < .001$, *Cohen's d* = 1.11), whereas there was no difference between the congruent and incongruent trials in the misaligned condition ($t_{(19)} = 1.878$, $p = .076$, *Cohen's d* = 0.5).

For children, the main effect of congruency showed that the sensitivity in the congruent trials was higher than in the incongruent trials ($F_{(1,21)} = 39.165$, $p < .001$, $\eta_p^2 = .651$). Additionally, the Congruency × Alignment interaction was also significant ($F_{(1,21)} = 9.037$, $p = .007$, $\eta_p^2 = .301$). The post-hoc paired-samples *t*-tests revealed in the aligned condition, the sensitivity in the congruent trials was higher than in the incongruent trials ($t_{(21)} = 5.495$, $p < .001$, *Cohen's d* = 1.7); in the misaligned condition, the sensitivity in the congruent trials was also higher than in the incongruent trials ($t_{(21)} = 2.865$, $p = .009$, *Cohen's d* = 0.5).

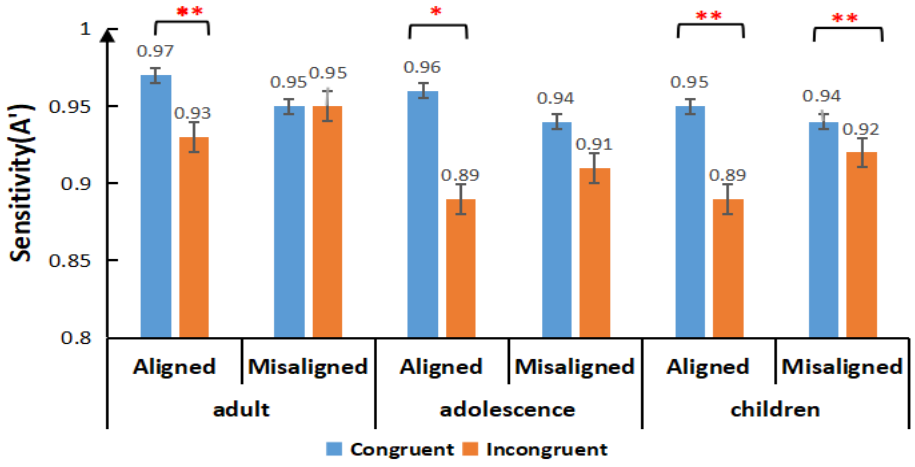

Figure 4. Mean sensitivity (A') for the congruent and incongruent trials as a function of Alignment (aligned/misaligned) for each group. (* p < 0.05; ** p < 0.01; *** p < 0.001)

1.2 Reaction time in each group

A 2 × 2 repeated-measures ANOVA was performed on the correct response times in each group (see Figure 5). For adults, there was a significant main effect of congruency ($F_{(1,24)} = 20.472$, $p < .001$, $\eta_p^2 = .460$), whereby the RTs in the congruent trials was faster than in the incongruent trials. A Congruency × Alignment interaction was found($F_{(1,24)} = 14.433$, $p < .001$, $\eta_p^2 = .376$). The post-hoc paired-samples *t*-tests revealed in the aligned condition, the RTs in the congruent trials was faster than in the incongruent trials ($t_{(24)} = 5.103$, $p < .001$, *Cohen's d* = 0.49), whereas the difference between them was not significant in the misaligned condition ($t_{(24)} = 0.708$, $p = .486$, *Cohen's d* = 0.06).

For adolescents, the main effect of alignment was significant ($F_{(1,19)} = 18.531$, $p < .001$, $\eta_p^2 = .494$), with the RTs in the aligned condition faster than in the misaligned condition. There was an interaction between Congruency and Alignment ($F_{(1,19)} = 24.619$, $p < .001$, $\eta_p^2 = .564$). The post-hoc paired-samples *t*-tests revealed that in the aligned condition, the RTs in the congruent trials was faster than in the incongruent trials ($t_{(19)} = 4.083$, $p = .001$, *Cohen's d* = 0.33); however, in the misaligned condition, there was no difference between them ($t_{(19)} = 1.788$, $p = .090$, *Cohen's d* = 0.11).

For children, the significant main effect of alignment revealed that the RTs in the aligned condition was faster than in the misaligned condition ($F_{(1,21)} = 17.920$, $p < .001$, $\eta_p^2 = .460$). There was an interaction between Congruency and Alignment($F_{(1,21)} = 7.031$, $p = .015$, $\eta_p^2 = .251$). The post-hoc paired-samples *t*-tests revealed in the aligned condition, the RTs in the congruent trials was faster than in the incongruent trials ($t_{(19)} = 2.975$, $p = .007$, *Cohen's d* = 0.23), whereas no difference was found between them in the misaligned condition ($t_{(19)} = 1.045$, $p = .308$, *Cohen's d* = 0.08). ($F_{(1,21)} = 7.031$, $p = .015$, $\eta_p^2 = .251$).

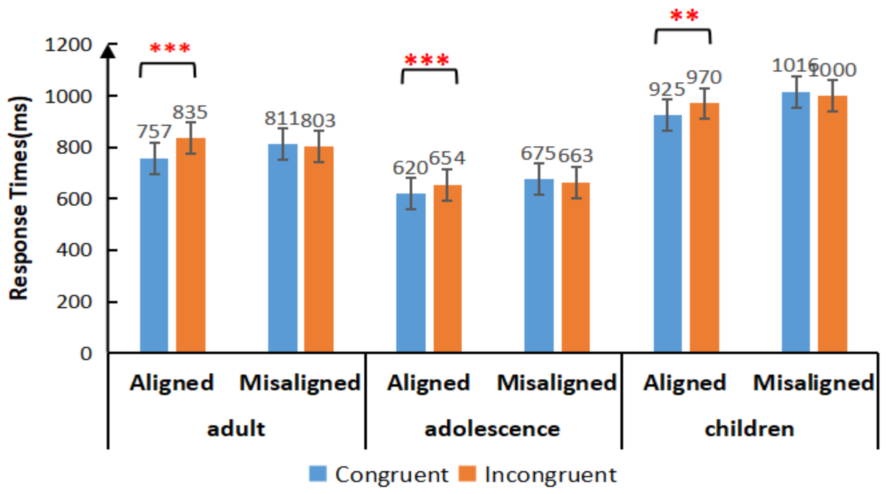

Figure 5. The correct reaction times (RT) for the congruent and incongruent trials as a function of Alignment (aligned/misaligned) for each group. (* p < 0.05; ** p < 0.01; *** p < 0.001)

2 The results in the first manuscript

In the first manuscript, three age groups were recruited: 25 children, 25 adolescents and 16 young adults (three children and five adolescents were excluded from further analysis). As suggested by one of the reviewers, in the revised manuscript, we added 9 adult participants. So we also reported the results of the first manuscript (22 children, 20 adolescents and 16 young adults) as followed:

Sensitivity (A')

The analysis revealed a significant main effect of Congruency ($F_{(1,55)} = 47.917$, $p <$ 0,.001, $\eta_p^2 = .466$), whereby sensitivity in the congruent condition was significantly higher than in the incongruent condition. There was a significant main effect of Group ($F_{(2,55)} = 4.298$, $p = .018$, $\eta_p^2 = .135$). Moreover, a significant interaction was found between Congruency and Group ($F_{(2,55)} = 4.982$, $p = .010$,$\eta_p^2 = .153$). The post-hoc independent-samples t-tests revealed that there were no significant differences among the groups in the congruent condition. Critically, in the incongruent condition, the face sensitivities of adults were significantly greater than those of adolescents ($t_{(34)} = 3.121$, $p = 0.004$, *Cohen's d* = 1.07) and children ($t_{(36)} = 4.494$, $p < 0.001$, *Cohen's d* = 1.45). There was no difference between the face sensitivities of adolescents and children, which indicates the presence of continuous development of face processing ability from adolescence to young adulthood.

Importantly, the results revealed a significant interaction between Congruency and Alignment ($F_{(1,55)} = 25.15$, $p < .001$,$\eta_p^2 = .314$). Further analysis using paired-samples t-tests revealed that in the aligned condition, sensitivity was significantly greater in the congruent trials than in incongruent trials ($t_{(57)} = 7.967$, $p < 0.001$, *Cohen's d* = 1.21). In the misaligned condition, sensitivity was marginally significantly greater in the congruent trials than in the incongruent trials ($t_{(57)} = 1.968$, $p = 0.054$, *Cohen's d* = .27). Importantly, there were no three-way interactions.

Reaction time

The results revealed a significant main effect of Congruency ($F_{(1,55)} = 9.771$, $p = .003$,$\eta_p^2 = .151$), whereby the RTs of congruent trials were faster than those of incongruent trials. The main effect of Alignment was also significant ($F_{(1,55)} = 32.632$, $p < 0.001$,η_p^2= .372); the RTs for aligned faces were faster than those of misaligned faces. In addition, a significant main effect of Group was found ($F_{(2,55)} = 22.196$, $p <$ 0.001,$\eta_p^2 = .447$).

The interaction between Alignment and Group was significant ($F_{(1,55)} = 3.433$, $p =$ 0.399,η_p^2= .111). The post-hoc *t*-tests revealed that in the aligned condition, the RTs of adolescents were significantly faster than those of adults ($t_{(34)} = 3.912$, $p < 0.001$, *Cohen's d* = 1.27) and children ($t_{(40)} = 6.427$, $p < 0.001$, *Cohen's d* = 2.02), whereas the RTs of children were slower than those of adults ($t_{(36)} = 2.296$, $p = 0.028$, *Cohen's d* = .77). Furthermore, in the misaligned condition, the RTs of adolescents were also significantly faster than those of adults ($t_{(34)} = 3.703$, $p < 0.001$, *Cohen's d* = 1.21) and children ($t_{(40)} = 6.71$, $p < 0.001$, *Cohen's d* = 2.11), whereas the RTs of children were slower than those of adults ($t_{(36)} = 2.958$, $p = 0.005$, *Cohen's d* = .99).

Importantly, similar to the result of sensitivity (A'), Congruency × Alignment was also significant ($F_{(1,55)}$ = 25.115, p < 0.001, η_p^2= .313). The post-hoc *t*-tests revealed that in the aligned condition, the RTs in the congruent trials were faster than those in the incongruent trials ($t_{(57)}$ = 5.679, p < 0.001, *Cohen's d* = .23); and in the misaligned condition, the RT of the congruent trials was slower than that of the incongruent trials ($t_{(57)}$ = 2.024, p = 0.048, *Cohen's d* = 0.07).

There were two slightly different results between the first manuscript and the revised manuscript whereas it did not affect the main results of the experiment:
(1) **sensitivity A'**: In the post-hoc *t*-tests of the interaction between Congruency and Alignment, the difference between congruent and incongruent trials in the misaligned condition was marginally significant in the first manuscript ($t(57)$ = 1.968, p = 0.054), which was significant in the revised manuscript ($t(66)$ = 2.020, p = .047).
(2) **Reaction time**: In the post-hoc *t*-tests of the interaction between Congruency and Alignment, although the difference between congruent and incongruent trials in the misaligned condition was significant in the first manuscript ($t(57)$ =2.024, p = 0.048), it was marginally significant in the revised manuscript ($t(66)$ = 1.717, p = .091).
